# The association between diet price and diet quality among Australian adults participating in the 2020 International Food Policy Study

**DOI:** 10.1017/S0007114525105461

**Published:** 2025-11-28

**Authors:** Carina Mammone, Todd Wallace, Christine White, Kathryn Backholer, Clara Gomez-Donoso, Gary Sacks, Adrian J. Cameron, Laura Alston, David Hammond, Christina Zorbas

**Affiliations:** 1 Global Centre for Preventive Health and Nutrition, Institute for Health Transformation, Deakin University, Geelong, VIC, Australia; 2 School of Public Health Sciences, University of Waterloo, Waterloo, ON, Canada; 3 Deakin Rural Health, Deakin University, Warrnambool, VIC, Australia

**Keywords:** Food price, Diet price, Diet quality, Food security, Obesity prevention

## Abstract

This cross-sectional study examined the association between diet price and diet quality in a national sample of Australian adults (*n* 1956). Diet recall data from the 2020 International Food Policy Study were linked to a national food and beverage price dataset. Daily diet price was calculated by summing the median non-promotional prices of all foods and beverages recorded in diet recall data, priced per gram (or millilitre) and adjusted for edible portions. Diet quality was determined using the Australian Dietary Guideline Index 2013 (scored out of 115). Linear regression models tested the association between the diet price (per dollar and per ten-dollar increments) and diet quality, adjusted for education, age and sex. A positive association was observed, where diet quality increased by 0·09 units (95 % CI 0·05, 0·14) for every $AU 1 increase in diet price. Daily diet price explained approximately 8 % of the variation in diet quality across the sample (*R* ^2^ = 0·08). When categorised in ten-dollar increments, participants with diet prices < $AU 10/d had a lower mean diet quality score (51·96) compared with all other diet price categories, 5–6 points lower than those whose diet was > $30/d. Diet price appeared to be a modest yet significant determinant of diet quality for Australian adults in 2020. Additional analyses are needed to investigate these associations during recent food inflation. As diet quality appears to be lowest for people who spend the least on food, government action to increase priority communities’ food budgets may help improve the nutritional quality of population diets.

Diet risks are leading contributors to preventable chronic diseases and include suboptimal dietary intakes that are low in whole grains, fruits, vegetables, nuts and seeds, milk, calcium, fibre and seafood and high in processed and red meats, sodium, trans fats and sugar-sweetened beverages^(1)^. In 2019, nearly 8 million deaths and 190 million disability-adjusted life years were attributable to diet risks worldwide^(1)^. There is academic consensus that modern, globalised food systems have driven the increased consumption of diets that promote weight gain and chronic diseases^(2)^. Diet quality is a commonly used index for measuring the extent to which an individual’s food consumption patterns reflect healthy eating guidelines^(3)^. Analyses using multiple diet quality indices suggest that adults’ diet quality is modest globally (i.e. a global mean of 40 out of 100), with similar patterns across high-income countries^(4)^. In Australia, the Commonwealth Scientific and Industrial Research Organisation routinely monitors diet quality in a convenience sample, finding a mean diet quality score of 55 out of 100 between 2015 and 2023^(5)^. Across various country contexts, socio-economic position (SEP) has also been associated with diet quality, with evidence suggesting that people of lower SEP are more likely to have lower quality diets compared with those of higher SEP^(6,7)^. Swiss researchers, for example, found that diet quality explained 22–36 % of socio-economic inequalities in obesity^(8)^.

Studies have found that, across different settings, food and beverage prices and affordability are perceived as being leading determinants of inequities in healthy diets^(9–11)^. Recognising the importance of monitoring food and beverage prices to inform public health policy priorities, methods have been pioneered by the International Network for Food and Obesity/Non-communicable Diseases Research, Monitoring and Action Support – a researcher-led network that is active in 65+ countries^(12)^. One method produced by the International Network for Food and Obesity/Non-communicable Diseases Research, Monitoring and Action Support includes the Australian Standardised Affordability and Pricing (ASAP) protocol^(13)^. The ASAP protocol measures the bi-weekly cost of foods and beverages forming a healthy diet (aligned with the Australian Dietary Guidelines) for a four-person reference household, compared with the cost of foods and beverages included in a current (less healthy) diet^(13)^. Over the last 8 years, the prices of these two diets have only been estimated in selected areas in Australia due to a lack of monitoring resources and comprehensive food consumption data. The ASAP body of evidence generally indicates that a healthy diet can be less expensive than the current (less healthy) diet that populations typically consume, although both diets tend to be unaffordable for people living on low incomes^(14,15)^.

Several additional studies using larger food price datasets from various countries have been conducted to more comprehensively investigate the association between the price and affordability of foods and diet quality. For example, in the USA, Spain and Japan, higher quality diets have been found to be more expensive than lower quality diets^(16–18)^. Such country-specific evidence is lacking in many other parts of the world, including in Australia. Despite the importance of food prices for the diet-related health of populations, we have limited evidence to support our understanding of how the price of diets varies as diet quality changes. Localised research can support the prioritisation of national-level policies to address food price and affordability from a public health standpoint – especially in light of recent price hikes and economic, social and health crises that have garnered political attention in Australia and abroad^(19,20)^.

To fill this knowledge gap in Australia, we aimed to assess the association between diet price and diet quality (calculated to represent daily consumption), using recent food price and diet consumption data. In line with considerable research that has found healthy diets to be less expensive than current unhealthy diets in some regions of Australia, but still unaffordable for people of low SEP, our hypothesis was that we would also find that higher quality diets would be less expensive than lower quality diets^(13,21,22)^.

## Methods

### Study design

A cross-sectional study was conducted using food and beverage consumption and price data from 2020. Australian diet recall data from the 2020 International Food Policy Study (IFPS) survey were matched to food and beverage prices sourced from the PriceTracker database. PriceTracker constitutes online food and beverage prices from the largest Australian food retailers (details below).

### Data sources

#### PriceTracker

PriceTracker is a database with weekly price data for over 20 000 unique food and beverage products from the two largest supermarkets (by market share) in Australia^(23)^. Price data collection is automated, using data from online supermarket websites that we have found to be comparable to data from retailers’ physical stores^(23)^. We used 1 week of food and beverage price data collected in November 2020 from the Australian supermarket with the highest grocery market share^(24)^, with the date chosen to correspond with the IFPS diet recall data collection.

#### International Food Policy Study (IFPS)

The IFPS is a cross-sectional survey repeated annually to investigate dietary patterns and the impact of national-level food policies across five countries, including Australia^(25)^. Data are collected via self-completed web-based surveys conducted annually in November to December with adults. Participants were recruited from a Nielsen Consumer Insights Global Panel and their partners’ panels, using sampling targets for age, sex and education to facilitate recruitment of a sample resembling the sociodemographic profile of the Australian population as closely as possible. Beginning in 2020, 24-h diet recall data were collected from IFPS participants in Australia using the Australian version of the validated Automated Self-Administered 24-H Dietary Assessment Tool (ASA24)^(26)^. To minimise the impact of potential underreporting of dietary intake, diet recall data were only included for male participants who, over a 24-h period, consumed between 850 kcal–4000 kcal and female participants who consumed between 500 kcal–3500 kcal^(27)^. The diet recall data were also filtered to exclude participants with a recall completion time of under 5 min or who indicated that they consumed ‘less than usual’ over the 24-h period of completing the recall survey in comparison with their typical consumption. These strategies were used to address cases of underreporting in the analysis.

### Sample

Our sample was from the Australian arm of the IFPS 2020 wave. Respondents were screened for eligibility with quota requirements based on age group and sex. To be eligible, individuals needed to be aged 18–100 years and live in Australia^(25)^. Email invitations were sent to a random sample of eligible panellists^(25)^. Respondents provided consent prior to survey completion and received remuneration in accordance with their panel’s existing reward structure. A full description of the study methods can be found in the International Food Policy Study: Technical Report – 2020 Survey (Wave 4)^(25)^.

#### Exposure variable: daily diet price

The IFPS and PriceTracker data were imported into STATA(SE) 17 Statistical Software^(28)^ and cleaned for analysis. Only participants who fully completed an ASA24 dietary recall were included in our analysis. At the item level, food and beverages in the ASA24 and PriceTracker datasets were separately coded using Australian Food and Nutrient Database food codes^(29)^. The PriceTracker dataset was restricted to only those food codes listed in the ASA24 diet recall dataset. These food and beverage items were then priced per gram (including millilitres where equivalent and considering the different amounts of ingredients in recipes) using the median non-promotional prices of products for each food code multiplied by their edible portions (provided by the Australian Food and Nutrient Database)^(29)^. Once all food and beverage items within the diet recalls were priced, they were summed to create a daily diet price for each participant.

#### Outcome variable: diet quality

Diet quality was determined using the Australian Dietary Guideline Index 2013 developed by Thorpe *et al.* ^(3)^. This index reflects a score out of a maximum of 130 and reflects the most recent dietary guidelines in Australia. Each participant’s diet was scored according to nutrient-based criteria (i.e. measuring diet variety and intakes of vegetables and fruits, cereals, lean meat and alternatives (including the proportion of lean meats and alternatives to total meat and alternatives per day), dairy products and alternatives, fluid, saturated fat, added sugars), where each criterion is scored from 0 to 5 or 0 to10 (see online Supplementary Table S1 for components and scoring methods of the Dietary Guideline Index 2013)^(3)^. We modified the Dietary Guideline Index to a maximum score of 115 (from the original maximum score of 130) to accommodate missing data on salt intake and the exclusion of alcohol, with a higher score reflecting increased compliance with the Australian Dietary Guidelines. Alcohol was not included in our main diet price and diet quality analyses, as it has a disproportionate effect on dietary costs and has been excluded in similar studies^(30,31)^.

#### Covariates

Age (continuous), sex (‘female’ or ‘male’) and educational attainment (‘low’: completed year 12 or lower; ‘medium’: completed trade certificate/diploma or some university (below the bachelor’s level); or ‘high’: bachelor’s degree or higher) were obtained from the IFPS 2020 survey data and included as confounders in multivariable analyses. Age, sex and educational attainment are independently associated with both diet choices and the amount of money available for individuals to spend on foods and beverages. Other sociodemographic variables that have an influence on diet quality and price were described but not treated as confounders due to collinearity with education. This included Aboriginal status (yes/no), language spoken at home (only English/language other than English), equivalised household annual income ($AU based on income categories) and using postcode to identify area-level SEP based on the Index for Relative Socioeconomic Disadvantage^(32)^ and rurality based on a Monash Modified Model^(33)^.

### Statistical analyses

Diet quality and diet price are descriptively summarised as means and medians (i.e. to account for skewed price data), respectively. Sociodemographic differences in diet quality and diet price were tested using ANOVA and Kruskal–Wallis tests. An unadjusted linear regression model was fitted to the data first to estimate associations between daily diet price (independent variable) and diet quality (dependent variable). This was followed by a model including potential confounders (age, sex and educational attainment) that were significantly associated with diet price in unadjusted models. A linear regression model was also fitted with diet price entered as a categorical variable (i.e. in $AU 10 intervals) to organise the data in more meaningful terms for analysis and interpretation.

Data were weighted with post-stratification sample weights constructed using a raking algorithm with population estimates from the census based on age group, sex at birth, education, region and ethnicity (defined as speaking a language other than English or speaking only English in the home). Finally, the adjusted model was stratified by sex and age to investigate potential differences in the association between diet price and quality.

Sensitivity analyses were conducted to understand the association between diet price and diet quality when including alcohol products in both variables, with the view to clarify whether alcohol skewed any of the observed associations^(34)^. Additional sensitivity analyses were performed to identify the influence of total energy (kcal) on the association between diet price and diet quality. Statistical significance was defined as *P*< 0·05 for all analyses.

### Ethics

Data collection for the IFPS received ethics clearance through the University of Waterloo Research Ethics Committee (ORE# 30829).

## Results

### Sample characteristics

A total of 187 229 people were invited to participate in the IFPS study in Australia, and 5500 people attempted the ‘core’ IFPS survey. Of those, 1211 were excluded from the sample due to data quality concerns (e.g. missing region data; invalid response to a data quality question; completion of the ‘core’ IFPS survey prior to the dietary recall in under 15 min). A further 1196 were excluded from the analytical sample due to an incomplete dietary recall. Among the remaining 3093 respondents, 1956 responses were deemed to be eligible to be included in this research, with the additional exclusion of 1137 participants whose diets could not be priced (*n* 4), who completed the diet recall in under 5 min (*n* 398), who reported eating less than usual (*n* 312), who had implausible energy intakes (*n* 415) or missing education level (*n* 8).

Table 1 summarises the characteristics of the 2020 IFPS ASA24 sample who completed a dietary recall and were included in this analysis (*n* 1956). As shown in Table 1, 44 % of the analytic sample had completed year 12 or lower, 32 % had completed a trade certificate/diploma or some university (below the bachelor’s level) and 24 % had a bachelor’s degree or higher. Approximately 2 % of the sample identified as Aboriginal or Torres Strait Islander, and 21 % spoke a language other than English in the home. Additionally, 32 % of individuals had an equivalised annual household income less than $26 665 (below the Australian poverty line of $31 060 for single persons)^(35)^, while 33 % of participants had an equivalised annual household income above the Australian median equivalised household income (∼$50 232 or over). There was a relatively even spread of participants across areas with different SEP. Most of the participants (73 %) lived in metropolitan areas (Monash Modified Model level 1), while a few (< 1 %) lived in remote communities (i.e. Monash Modified Model level 6 or 7). Compared with the final analytic sample, when including respondents who completed diet recalls but were excluded due to data quality concerns or missing education level (*n* 3093), the original sample had a higher proportion of males (44 % *v*. 49 %) but a lower proportion of younger people aged 18–34 (25 % *v*. 15 %) and people speaking a language other than English at home (21 % *v*. 13 %). All other characteristics were similar (see online Supplementary Table S2).

**Table 1.** Sociodemographic characteristics of the analytical sample, including weighted mean diet quality and median diet price summaries

	*n*	%	Mean diet quality index score (out of 115)	95 % CI	ANOVA: *F*-test statistic, *P*	Median daily diet price ($)	IQR	Kruskal–Wallis H test, *P*
Total	1956		54·41	53·78, 55·04	n/a	21·29	19·06	n/a
Sex at birth								
Male	884	44·22 %	53·12	52·16, 54·06	F = 12·52, *P*< 0·001	22·46	19·83	Chi2 = 15·73, *P* < 0·001
Female	1072	55·78 %	55·44	54·60, 56·28		20·31	17·79	
Age								
18–34	444	25·07 %	50·58	49·34, 51·81	F = 25·16, *P* < 0·001	20·94	17·48	Chi2 = 6·99, *P* = 0·221
35–44	294	16·52 %	50·89	49·23, 52·56		20·45	18·17	
45–54	275	14·71 %	53·7	52·25, 55·30		20·87	18·13	
55–64	492	23·79 %	57·71	56·43, 58·99		22·29	19·92	
65–74	339	15·18 %	58·43	56·95, 59·92		21·51	18·74	
75+	112	4·72 %	59·55	57·02, 62·07		21·68	19·39	
Educational attainment								
Low	898	43·92 %	54·21	53·27, 55·14	F = 0·15, *P* = 0·863	20·88	20·30	Chi2 = 0·710, *P* = 0·701
Medium	607	32·08 %	54·54	53·42, 55·65		21·60	18·43	
High	451	23·99 %	54·61	53·28, 55·96		21·40	17·76	
Aboriginal or Torres Strait Islander status								
Aboriginal or Torres Strait Islander	44	2·36 %	46·62	42·91, 50·33	F = 11·31, *P* < 0·001	25·10	22·84	Chi2 = 1·089, *P* = 0·297
Non-Aboriginal	1907	97·53 %	54·59	53·95, 55·23		21·13	18·86	
Language spoken at home								
Speak a language other than English at home	233	21·02 %	53·47	51·63, 55·31	F = 2·56, *P* = 0·078	19·45	15·06	F = 4·53, *P* = 0·104
Only speak English at home	1721	78·88 %	54·64	53·977, 55·31		21·59	19·32	
Equivalised household annual income ($AU)								
Less than 26 665	570	31·56 %	53·29	52·11, 54·47	F = 3·05, *P* = 0·048	21·55	19·68	F = 3·25, *P* = 0·197
26 665 to less than 50 232	650	35·20 %	54·73	53·63, 55·83		22·28	18·60	
Greater than 50 232	592	33·25 %	55·49	54·37, 56·61		21·08	19·66	
Area-level socio-economic position (Index for Relative Socioeconomic Disadvantage)								
1–2 (most disadvantaged)	359	17·40 %	53·36	51·95, 54·76	F = 1·19, *P* = 0·312	24·36	22·70	F = 9·09, *P* = 0·059
3–4	315	16·24 %	54·65	53·03, 56·27		20·61	17·57	
5–6	461	23·52 %	54·08	52·71, 55·46		20·56	16·84	
7–8	391	20·35 %	54·98	53·638, 56·34		20·33	17·70	
9–10 (least disadvantaged)	430	22·48 %	54·86	53·53, 56·20		21·34	18·83	
Rurality (Monash Modified Model)								
1 (metropolitan areas)	1391	73·46 %	54·27	53·51, 55·04	F = 1·32, *P* = 0·246	21·45	18·77	F = 3·62, 0·729
2 (regional centres)	164	8·19 %	53·81	51·77, 55·85		20·31	17·71	
3 (large rural towns)	95	4·60 %	56·46	53·81, 59·12		22·26	18·25	
4 (medium rural towns)	36	1·61 %	58·99	54·29, 63·70		22·76	17·41	
5 (small rural towns)	238	11·34 %	54·95	53·22, 56·68		20·70	20·69	
6 (remote communities)	13	0·61 %	49·42	43·66, 55·18		30·35	36·17	
7 (very remote)	4	0·18 %	51·09	19·98, 82·20		25·73	24·91	

The mean diet quality score was 54·41 out of 115 (CI: 53·78, 55·04), and the median daily diet price was $AU 21·29 (IQR 19·06). Table 1 outlines mean diet quality scores and median prices (with sample weights) by sociodemographic characteristics. Diet quality was only significantly different according to sex, age, Aboriginal or Torres Strait Islander Status and equivalised household income. Diet price was only significantly different according to sex.

### Associations between daily diet price and diet quality

**Fig. 1.**
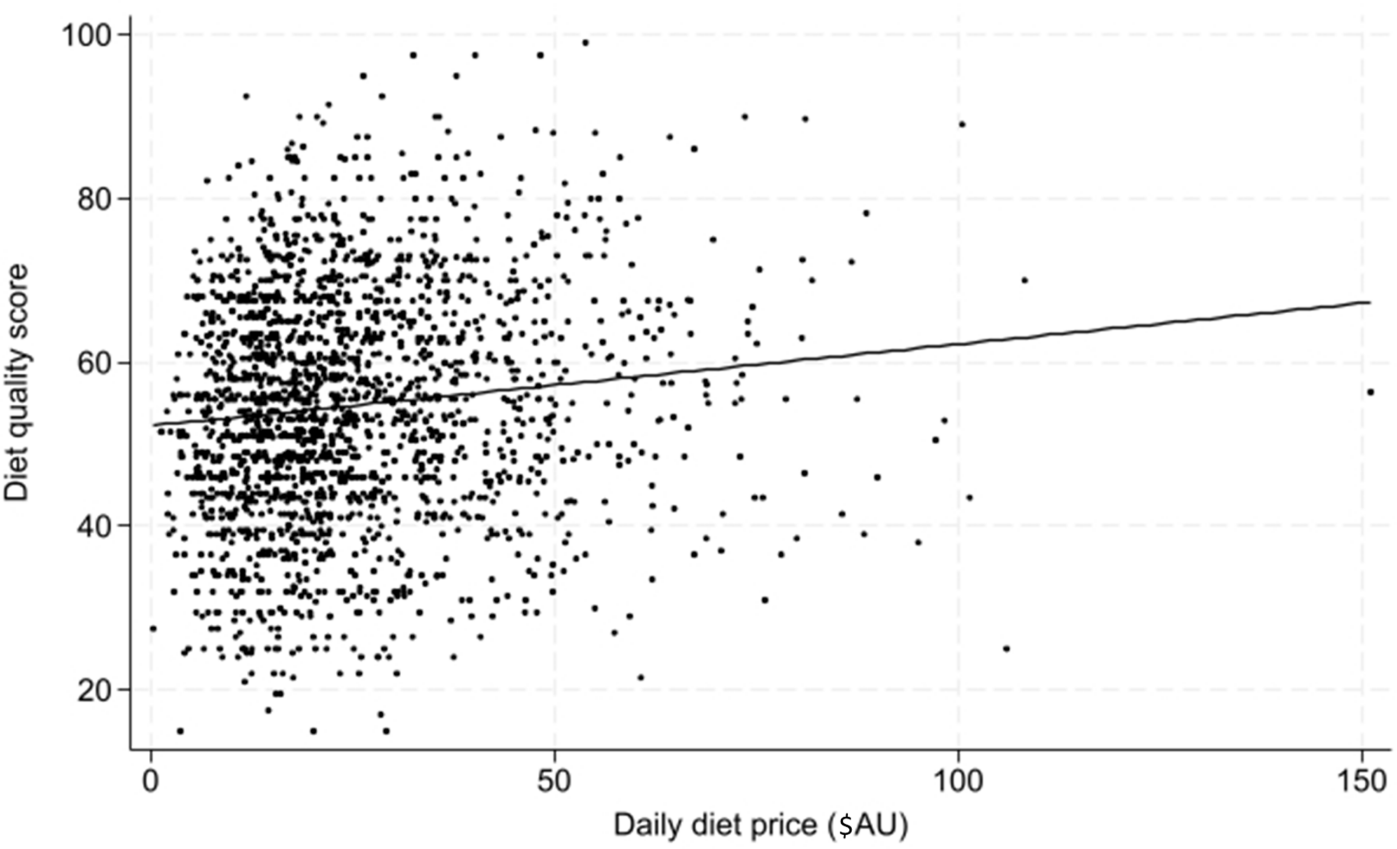
Scatter plot of adjusted association between daily diet price and diet quality.

In the adjusted regression model (adjusted for age, sex and education), daily diet price explained 8 % of the variance in diet quality (unadjusted *R* ^2^ = 0·01, adjusted *R* ^2^ = 0·08). The results shown in Fig. 1 indicate that diet price was positively associated with diet quality (*β* coefficient = 0·09; 95 % CI 0·05, 0·14; se = 0·02; *P*< 0·05).

Tables 2 and 3 show the association between daily diet price and diet quality when stratified by sex and age groups. When stratifying the adjusted regression model by sex, daily diet price explained 9 % of the variance in diet quality (adjusted *R* ^2^ = 0·09) for females. For males, daily diet price explained 6 % of the variance in diet quality (adjusted *R* ^2^ = 0·06). Daily diet price was positively associated with diet quality for both females (*β* coefficient = 0·12; 95 % CI 0·06, 0·17; se = 0·03; *P*< 0·05) and males (*β* coefficient = 0·07; 95 % CI 0·00, 0·14; se = 0·04; *P*< 0·05). When stratifying by age group, the association between diet price and diet quality was only significant for the 55–64 and 65–74 age groups, although the adjusted *R* ^2^ values lowered to 0·04 and 0·06 for these age groups, respectively.

**Table 2.** Associations between daily diet price and diet quality, stratified by sex

Age range	*n*	*β* coefficient	95 % CI	se	*P*	Adjusted *R* ^2^
Females	1072	0·12	0·06, 0·17	0·03	0·00	0·09
Males	884	0·07	0·00, 0·14	0·04	0·05	0·06

**Table 3.** Associations between daily diet price and diet quality, stratified by age groups

Age range	*n*	*β* coefficient	95 % CI	se	*P*	Adjusted *R* ^2^
18–34	444	0·06	–0·04, 0·15	0·05	0·23	0·01
35–44	294	0·10	–0·05, 0·25	0·08	0·19	0·05
45–54	275	0·07	–0·05, 0·20	0·06	0·24	0·06
55–64	492	0·10	0·03, 0·18	0·04	0·01	0·04
65–74	339	0·11	0·02, 0·20	0·05	0·02	0·06
75+	112	0·16	–0·02, 0·34	0·09	0·09	0·10

Table 4 shows the results of using daily diet price as a categorical variable in fully adjusted models. Statistically significant differences (*P*< 0·05) were observed for diet quality across each daily diet price category compared with the lowest daily diet price category (≤ $AU 10). The mean diet quality score of people who spent ≤ $AU 10 per d was 51·69. Participants in the highest diet price category (who spent > $50 per d) also had the highest mean diet quality score, with a mean diet quality score of 58·09 (95 % CI 55·58, 60·61; *P*< 0·05), which was 6·40 points higher compared with people with the lowest daily diet prices.

**Table 4.** Mean diet quality score by daily diet price category (according to adjusted regression model)

Daily diet price category	Mean diet quality score	95 % CI	*P*	*β* regression coefficients	95 % CI	*P*
Less than or equal to $10 (*n* 271)	51·69 (reference)			0 (reference)		
Between $11 and $20 (*n* 653)	53·60	52·52, 54·68	*P* = 0·027	2·17	0·24, 4·10	*P* = 0·027
Between $21 and $30 (*n* 453)	55·32	54·00, 56·63	*P* < 0·001	3·70	1·63, 5·77	*P* < 0·001
Between $31 and $40 (*n* 279)	57·67	55·96· 59·39	*P* < 0·001	5·96	3·68, 8·25	*P* < 0·001
Between $41 and $50 (*n* 151)	55·21	52·94, 57·47	*P* = 0·003	4·34	1·45, 7·23	*P* = 0·003
Greater than $50 (*n* 148)	58·09	55·58, 60·61	*P* < 0·001	5·64	2·53, 8·74	*P* < 0·001

### Sensitivity analysis

Sensitivity analyses testing the strength of the association between daily diet price and diet quality after including alcohol slightly attenuated the beta coefficient (*β* coefficient = 0·06; 95 % CI 0·03, 0·08; se = 0·01; *P*< 0·05). In this adjusted sensitivity analysis model, daily diet price explained 6 % of the variance in diet quality (adjusted *R* ^2^ = 0·06). In a model testing the strength of the association between diet price and diet quality, the inclusion of total energy (kcal) in the model did not adjust the model fit (adjusted *R* ^2^ = 0·08). The inclusion of total energy (kcal) slightly attenuated the beta coefficient (*β* coefficient = 0·08; 95 % CI 0·03, 0·13; se = 0·03; *P*< 0·05).

## Discussion

This study aimed to comprehensively quantify the association between Australian adults’ daily diet prices and their diet quality. We found a positive association, where for every $AU 10 increase in diet price, the diet quality score increased by approximately 1·0 units (on a scale with a maximum of 115 points that indicated compliance with the Australian Dietary Guidelines). Overall, diet price explained approximately 8 % of the variability in diet quality across our sample of Australian adults. Although the strength of the association between price and diet quality was modest, when broken down, the positive relationship likely highlights the inequitable influence of food prices on diet quality across different populations. For example, the association was slightly stronger for females than for males. Moreover, respondents whose daily diet price was more than $50 had the highest mean diet quality score, which was 6·40 points higher than those in the lowest daily diet price category (≤ $AU 10). This difference of six points could represent a meaningful change in dietary patterns, such as an additional serving of fruits or vegetables or a reduced intake of less healthy foods with excess saturated fat or added sugars.

The moderate average diet quality of our sample (mean Dietary Guideline Index score: 54 out of 115) is somewhat aligned with recent estimates by the Commonwealth Scientific and Industrial Research Organisation in Australia. The Commonwealth Scientific and Industrial Research Organisation reported a mean diet quality score of 55 out of 100 using a different validated diet quality tool across a convenience sample of 235 268 adults between 2015–2023^(5)^. These two diet quality scores are not directly comparable due to the different scoring tools (where we examined fewer criteria) and samples (where our sample is smaller but more representative of the Australian population). Sociodemographic differences in our diet quality findings (which should be interpreted with consideration of our small sub-group sample sizes) also support existing literature that suggests that diet intakes are often lower quality among people who identify as Aboriginal and/or Torres Strait Islander^(36)^, are on the lowest incomes^(37)^ and reside in lower socio-economic^(37)^ and remote areas^(38)^. In our study, these populations (except for households with the lowest incomes) also tended to have higher diet prices and lower diet quality compared with the overall sample average. In contrast, females in our sample had higher quality diets than males but lower diet prices. This translated into stronger associations between diet price and diet quality for females than for males. While we cannot explain the causal mechanisms behind these differences, females have previously been found to eat more vegetables and less discretionary foods than males^(39)^, and different dietary components may be associated with different costs^(14)^. Similar explanations could also be applied to the age-related differences we observed^(39)^.

Few studies globally have examined the association between diet quality and diet price using population-based diet surveys and large price datasets, possibly due to limited access to appropriate data. A 2014 study testing the association between daily diet price and diet quality in the USA found similar results to ours^(40)^. In that study, the price per gram for each food and beverage item reported in the survey (excluding alcoholic beverages) was estimated using the 2003–2004 Center for Nutrition Policy and Promotion Food Prices Database^(40)^. The regression coefficient for daily diet price was 0·065 (*P*< 0·01), adjusted for age, sex and income, among other demographic factors and health behaviours, which is similar to the *β* regression coefficient found in our Australian study^(40)^. A US study from 2010 investigating the association between food prices and energy content found a slight positive association (*P*< 0·01, adjusted *R* ^2^ = 0·38), also finding that the association varied across macronutrient groups^(41)^. While each megajoule of energy per gram (MJ/g) of protein increased the price of foods by USD$3·26 (*P*< 0·01), using the same metric, carbohydrates reduced food prices by USD$0·38, while fats had no effect on food price^(41)^. In the UK, another study (2018) examining the association between individual daily diet price and compliance with the UK Government dietary guidelines found that diets meeting the recommendations were 3–17 % more expensive than diets that did not meet the recommendations^(42)^.

In Australia, no previous research has specifically examined the relationship between daily diet price and diet quality using a national dataset with individual dietary intake data. Smaller-scale studies using the ASAP method have measured differences in the cost of two standard diets across regions and household compositions (e.g. a family of four, single households, etc.). Such research has found that a hypothetical household diet that complies with the Australian Dietary Guidelines is less expensive than a diet based on current, less healthy consumption patterns^(13,21,22)^. While a hypothetical healthy diet can be cheaper than one type of less healthy diet, our findings show that there is a spectrum of variability in diet quality according to diet price when measuring real-world population intakes across a national sample. When assessing a variety of relatively healthier diets (i.e. not an optimal healthy diet) that Australian adults reported consuming in our study, these were, on average, more expensive than the less healthy diets reported.

Another key finding in existing ASAP literature is that both a hypothetical healthy diet and a less healthy diet are unaffordable for people in Australia on the lowest incomes^(14,15,43)^. We have previously estimated that healthy diets cost approximately $600 per fortnight or 25–30 % of the available spending money for families on low incomes (thresholds that have been found to be indicative of food stress)^(14)^. It is important to note that our analyses do not involve comparable metrics with these prior studies (i.e. we did not price an optimal healthy diet as a proportion of household income but rather looked at diet price as a predictor of diet quality). Nevertheless, we segmented the analysis into different diet price categories to help interpret our observed associations for people with different food budgets. In these scenarios, we found that people who spent less on their diets (≤ $AU 10/d = $140/fortnight) had the lowest diet quality. This is not surprising, reiterating suggestions that adequate incomes and food budgets, especially among low-income households, are important predictors of diet quality^(15)^. Moreover, if the price of optimal healthier diets exceeds people’s small food expenditure in the real world, achieving them may not be realistic for everyone.

### Implications for policy and research

Governments across Australia and internationally are increasingly recognising the impact of food prices, food affordability and food insecurity (especially following the coronavirus disease 2019 pandemic and cost-of-living crises) on population health. Importantly, for priority populations on low incomes, increasing government income support can provide more funds to spend on food, which is important for achieving food security and may also improve diet quality^(44,45)^. Policy interventions can also focus on lowering the prices of foods and beverages. For example, in Greece, the government implemented price caps on a basic basket of foods and other essential goods, alongside the threat of financial penalties for multinational companies that failed to lower their prices^(46)^. Additional research is needed to evaluate the long-term effectiveness of these recent food policies and other social measures for improving food prices, affordability and diet quality.

To further inform the prioritisation of evidence-based policy responses that promote equitable access to healthy, affordable diets, policymakers can utilise available food consumption and food price data to routinely monitor diet prices across communities. Our study advances this understanding of best practice approaches for measuring and monitoring diet prices, affordability and quality, over time, both within Australia and internationally. By leveraging large datasets with individual diet data from a national sample, our integrated approach offers an efficient and statistically rigorous way of monitoring the prices of real population diets, complementing previous approaches to monitoring optimal hypothetical diet costs and their affordability.

Moreover, given the observed association between diet price and diet quality in our study, and the important role of social factors in improving diet affordability and diet quality, future research should utilise large datasets to longitudinally examine other environmental and upstream factors contributing to diet quality over time. Notably, since 2020, global food prices have increased due to coronavirus disease 2019 supply chain disruptions, global conflict and climate events^(47)^, warranting ongoing analyses to understand whether diet prices have become a stronger predictor of diet quality across populations in recent years. Research using large datasets could also specifically examine how retailer practices, market concentration and supply chain factors affect food prices and diet quality^(47)^. In any case, future analyses should maintain an explicit focus on assessing how various upstream factors impact different populations, including those living in regional areas, Aboriginal and Torres Strait Islander communities and families experiencing financial hardship.

### Strengths and limitations

This study provides a population-based analysis of the relationship between diet price and diet quality using ASA24 diet recall data collected from Australian adults in 2020^(25)^ and shows a modest association, in line with global research^(40–42)^. The findings offer an alternative view from previous Australian research, which has indicated that a hypothetical healthy diet can be less expensive than a typical unhealthy diet^(14,15)^. Our study provides a novel contribution showing nuanced variations in diet quality across different diet price points. The findings highlight that diet prices alone do not fully explain diet quality, warranting additional attention to other key drivers of diet quality. However, due to the cross-sectional nature of our study, causal inferences should not be made, and future longitudinal research is required.

Although IFPS data are weighted to ensure the sample is representative of the age and sex distribution in each state and nationally, it may not be truly nationally representative given that respondents are all part of a market research panel. For instance, people who do not have access to the internet (even though internet penetration is 88 % in Australia)^(48)^ or who do not speak English are effectively excluded. While incomplete responses are a known limitation of diet recall data, we aimed to manage this by restricting the analysis to only those who report plausible energy intakes. These exclusions were necessary to strengthen the quality of the analysis by reducing outliers and incomplete data, but this exclusion criterion reduced the sample size from 3093 to 1956. Our analytical sample had a lower proportion of males and higher representations of young people than the full survey sample, with differences accounted for in stratified analyses. It is unclear what impact the higher representation of people speaking a language other than English at home in our sample may have had on the analysis; however, this may have increased the relevance of the findings for traditionally underrepresented groups. All other sociodemographic factors were comparable to the original sample.

In terms of the PriceTracker data, we utilised an objective data collection method by automatically collecting and storing food and beverage product prices from online supermarkets weekly, thus reducing measurement bias^(23)^. Zorbas *et al.* determined the reliability of using prices listed on the websites of major grocery retailers in Australia to measure the prices of products in-store in cities and inner regional areas^(23)^. Despite being a reliable measure of prices in Australia’s major supermarkets, not all Australians have access to major supermarkets, particularly those in rural or remote areas, and various pricing strategies are often used to discount items, often more so for less healthy foods and beverages^(49)^. Future research could aim to include food and beverage price collection in rural and remote areas and incorporate price promotions to refine our estimates.

### Conclusions

Using diet consumption and food price data from 2020, we estimated the prices of real-world diets among a national sample of Australian adults for the first time. We found that as the price of a person’s diet increases, there was a modest but statistically significant increase in the quality of their diet. Diet quality was lowest for people whose diets costed the least. Despite this, the large variability in both the price and quality of diets in Australian adults means that a greater understanding of their relationship and other interrelated factors is required, especially in the context of recent food price inflation.

## Supporting information

Mammone et al. supplementary material Mammone et al. supplementary material

## Data Availability

Data used in this study are not publicly available but can be accessed by contacting the research team and obtaining the required approvals from the data custodians.

## Supplementary material

For supplementary material/s referred to in this article, please visit https://doi.org/10.1017/S0007114525105461.
