# Supplementary material for: The association between diet price and diet quality among Australian adults participating in the 2020 International Food Policy Study
Source: Br J Nutr. 2025 Oct 14;134(10):863–71. doi: 10.1017/S0007114525105461 (PMC12795638; doi:10.1017/S0007114525105461)
Supplement: Mammone et al. supplementary material [file S0007114525105461sup001.docx]

# **SUPPLEMENTARY MATERIAL**

# **Table S1: Components and scoring methods of the revised Dietary Guideline Index (DGI-2013) developed by Thorpe et al (2016).**

**Source:** Thorpe MG, Milte CM, Crawford D, McNaughton SA. A revised Australian Dietary Guideline Index and its association with key sociodemographic factors, health behaviors and Body Mass Index in peri-retirement aged adults. Nutrients. 2016;8(3):160.

| Dietary guideline | Indicator and description | Criteria for maximum score | Criteria for minimum score | Maximum score |
| --- | --- | --- | --- | --- |
| Guidelines for adequate intake | | | | |
| 1. Enjoy a wide variety of nutritious foods | Food variety: proportion of food from each of the 5 core food groups eaten at least one serve per week | 100% | 0% | 10 |
| 2. Plenty of vegetables | Total vegetable intake: servings of vegetables per day | 19–50 y: M ≥ 6, F ≥ 5 | 0 | 10 |
|  |  | 51–70 y: M ≥ 5.5, F ≥ 5 |  |  |
|  |  | > 70 y: M ≥ 5, F ≥ 5 |  |  |
| 3. Fruit | Total fruit intake: servings of fruit per day | ≥2 | 0 | 10 |
| 4. Grain (cereal) foods | Total cereal intake: servings of grains per day | 19–50 y: M ≥ 6, F ≥ 6 | 0 | 5 |
|  |  | 51–70 y: M ≥ 6, F ≥ 4 |  |  |
|  |  | >70 y: M ≥ 4.5, F ≥ 3 |  |  |
|  | Mostly wholegrain or high fibre cereals: Type of bread usually consumed | Wholemeal bread | White bread | 5 |
| 5. Lean meat and poultry, fish, eggs, nuts and seeds, and legumes/beans | Total meat and alternative: servings per day | 19–50 y: M ≥ 3, F ≥ 2.5 | 0 | 5 |
|  |  | 51–70 y: M ≥ 2.5, F ≥ 2 |  |  |
|  |  | >70 y: M ≥ 2.5, F ≥ 2 |  |  |
|  | Lean meat: proportion of lean meats and alternatives to total meat and alternatives per day | 100% | 0% | 5 |
| 6. Milk, yoghurt, cheese and/or their alternatives | Total dairy and alternative: servings per day | 19–50 y: M ≥ 2.5, F ≥ 2.5 | 0 | 10 |
|  |  | 51–70 y: M ≥ 2.5, F ≥ 4 |  |  |
|  |  | >70 y: M ≥ 3.5, F ≥ 4 |  |  |
| 7. Drink plenty of water | Total beverage intake: servings per day | M ≥ 10; F ≥ 8 | 0 | 5 |
|  | Water: proportion of water to total beverage intake per day | ≥50% | 0% | 5 |
| Guidelines to limit or moderate intake | | | | |
| 8. Limit intake of foods containing saturated fat, added salt, added sugars and alcohol | Limit discretionary foods | M ≤ 3; F ≤ 2.5 | M > 3; F > 2.5 | 10 |
| 9. Limit intake of foods high in saturated fat | Trim meat: trimming fat from meat | Usually | Never or rarely | 5 |
|  | Choose reduced-fat milk: type of milk usually consumed | Skim, low or reduced fat milk | Whole milk | 5 |
| 10. Small allowance of unsaturated oils, fats or spreads | Unsaturated spreads and oils: servings per day | 19–50 y: M ≤ 4, F ≤ 2 | M > 4; F > 2 | 10 |
|  |  | 51–70 y: M ≤ 4, F ≤ 2 |  |  |
|  |  | >70 y: M ≤ 2, F ≤ 2 |  |  |
| 11. Limit intake of foods and drinks containing added salt | Salt use: salt added during cooking | Never or rarely | Usually | 5 |
|  | Salt use: salt added during the meal | Never or rarely | Usually | 5 |
| 12. Limit intake of foods and drinks containing added sugars | Limit extra sugar: servings per day | M ≤ 1.5; F ≤ 1.25 | M > 1.5; F > 1.25 | 10 |
| 13. If you choose to drink alcohol, limit intake | Limit alcohol: servings per day | ≤2 | >2 | 10 |

**Table S2: Comparison of the sociodemographic characteristics between the included analytical sample and the original sample who completed diet recalls in the Australian 2020 International Food Policy Study**

|  | **Included sample**  **n (%)** | **Original sample**  **n (%)** |
| --- | --- | --- |
| Total | 1956 | 3093 |
| **Sex at birth** | | |
| Male | 884 (44.22%) | 1524 (49.27%) |
| Female | 1072 (55.78%) | 1569 (50.73%) |
| **Age** | | |
| 18-34 | 444 (25.07%) | 401 (15.14%) |
| 35-44 | 294 (16.52%) | 519 (19.59%) |
| 45-54 | 275 (14.71 %) | 452 (17.06%) |
| 55-64 | 492 (23.79%) | 684 (25.82%) |
| 65-74 | 339 (15.18%) | 448 (16.91%) |
| 75+ | 112 (4.72%) | 145 (5.47%) |
| **Educational Attainment** | | |
| Low | 898 (433.92%) | 1367 (44.38%) |
| Medium | 607 (32.08%) | 966 (31.36%) |
| High | 451 (23.99%) | 747 (24.25%) |
| **Aboriginal or Torres Strait Islander Status** | | |
| Aboriginal or Torres Strait Islander | 44 (2.36%) | 114 (3.69%) |
| Non-aboriginal | 1907 (97.53%) | 2974 (96.31%) |
| **Language** **spoken at home** | | |
| Speak a language other than English at home | 233 (21.02%) | 386 (12.52%) |
| Only speak English at home | 1721 (78.88%) | 2698 (87.48%) |
| **Equivalised Household Annual Income ($AU)** | | |
| Less than 26,665 | 546 (27.90%) | 879 (30.87%) |
| 26,665 to less than 50,232 | 649 (33.18%) | 977 (34.32%) |
| Greater than 50,232 | 617 (31.54%) | 991 (34.81%) |
| **Area-level Socio-Economic Position (Index for Relative Socioeconomic Disadvantage)** | | |
| 1-2 (most disadvantaged) | 359 (17.40%) | 543 (17.76%) |
| 3-4 | 315 (16.24%) | 557 (18.21%) |
| 5-6 | 461 (23.52%) | 683 (22.33%) |
| 7-8 | 391 (20.35%) | 607 (19.85%) |
| 9-10 (least disadvantaged) | 430 (22.48%) | 668 (21.84%) |
| **Rurality (Monash Modified Model)** | | |
| 1 (metropolitan areas) | 1391 (73.46%) | 2217 (72.83%) |
| 2 (regional centres) | 164 (8.19%) | 257 (8.44%) |
| 3 (large rural towns) | 95 (4.60%) | 143 (4.70%) |
| 4 (medium rural towns) | 36 (1.61%) | 57 (1.87%) |
| 5 (small rural towns) | 238 (11.34%) | 338 (11.10%) |
| 6 (remote communities) | 13 (0.61%) | 23 (0.73%) |
| 7 (very remote) | 4 (0.19%) | 9 (0.30%) |
